# Structural Alterations of Human Erythrocytes Induced by Minocycline

**DOI:** 10.3390/cells14221787

**Published:** 2025-11-14

**Authors:** Elena Baeva, Marina Holyavka, Valery Artyukhov, Maxim Kondratyev

**Affiliations:** 1 Normal Physiology Department, Voronezh State Medical University Named After N.N. Burdenko, Studencheskaya Str. 10, Voronezh 394036, Russia; elena.s.baeva@mail.ru; 2 Biophysics and Biotechnology Department, Voronezh State University, Universitetskaya sq. 1, Voronezh 394018, Russia; artyukhov@bio.vsu.ru; 3 Institute of Cell Biophysics, Russian Academy of Sciences, Institutskaya St. 3, Puschino 142290, Russia; ma-ko@bk.ru

**Keywords:** erythrocyte, cytoarchitectonics, minocycline, spatio-temporal organization, molecular interactions, scanning electron microscopy, hemoglobin

## Abstract

The non-antibacterial effects of the tetracycline antibiotic minocycline on human erythrocytes are currently under investigation. Our data indicate alterations in the surface structure of erythrocytes; the antibiotic promotes the redistribution of cellular transformational forms during preliminary in vitro incubation (1 h and 24 h) with the modifier. The degree of surface relief changes increases over time, leading to the formation of erythrocytes displaying outgrowths and ridges, spherulation, and “deflated ball”-shaped cells (after 1 day). These alterations are largely reversible, as washing the erythrocyte suspensions with a 1% bovine serum albumin solution reduces the number of echinocytes and irreversibly transformed spherocytes with spikes. Spectrophotometric analysis has shown that minocycline stabilizes the spatial organization of hemoprotein molecules, as it does not lead to increased methemoglobin formation in the samples over time. The antibiotic appears to bind primarily to amino acid residues within heme pockets, as confirmed by molecular docking. Our findings suggest a potential risk of reduced oxygen transport function in red blood cells when taking this antibiotic, highlighting the need to consider potential erythrocyte-related side effects during long-term minocycline therapy.

## 1. Introduction

**Figure 1:**
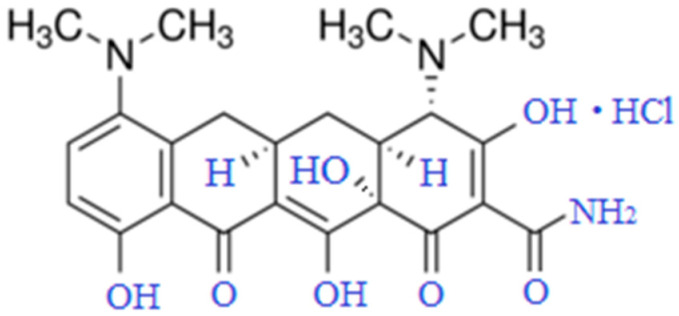
Minocycline hydrochloride (C_23_H_27_N_3_O_7_·HCl, Mr = 493.94 Da).

Tetracyclines, discovered in the mid-20th century, are widely used to treat inflammatory conditions of various origins. Long-term studies of their molecular structure and effects on prokaryotic cells have helped establish the primary mechanisms behind their antimicrobial action. Because eukaryotic organisms lack specific targets for tetracyclines, these antibiotics can affect a broad range of cell populations. For instance, tetracycline antibiotics have been found to induce changes in mitochondrial gene expression and metabolic activity [1]. Interest in tetracycline antibiotics continues to grow each year due to their range of non-antibacterial properties—immunomodulatory, antipyretic, analgesic, antitumor, and other activities [2,3,4,5]. Minocycline (MC) is a second-generation semi-synthetic tetracycline [6]. It has been used in therapy for over 30 years due to its antibacterial efficacy against both Gram-positive and Gram-negative bacteria (Figure 1). Certain structural modifications make MC more lipophilic than other drugs in its class. Its antibacterial effect works by inhibiting bacterial protein synthesis through binding to the 30S ribosomal subunit. In addition to its antimicrobial activity, MC exhibits anti-inflammatory, neuroprotective, antioxidant, immunomodulatory, and anti-apoptotic effects. It can also inhibit proteolysis, suppress angiogenesis, and reduce tumor metastasis, as demonstrated in various experimental models of non-communicable diseases [7,8,9,10,11,12,13,14].

The non-antibacterial effects of minocycline have been widely discussed in the scientific literature, particularly regarding its potential use in treating neurodegenerative diseases and its effects on blood cellular components [15,16,17,18,19]. Minocycline can exert antinociceptive effects, induce autophagy in various tumor types both in vitro and in vivo, and influence the pro-oncogenic TGF-β–NF-κB axis as well as the IKKα/β signaling pathway. This indicates a beneficial, context-dependent role in modulating cellular homeostasis and the pathogenesis of socially significant diseases. Minocycline can, in a dose-dependent manner, perform the following: inhibit HIF-1α protein expression and HIF-1 transcriptional activity; reduce VEGF expression, protein synthesis, and mTOR signaling while increasing phosphorylation of eIF2α; suppress the cytokines TNF-α, IL-6, IFN-γ, and the chemokines IL-8, IP-10, MCP-1, MIP-1α, MIP-1β, and eotaxin; significantly inhibit nuclear factor-κB alpha (IκBα) and IκB kinases (IKK)α/β; directly inhibit calcium influx through N-methyl-D-aspartate (NMDA) receptors, which reduces mitochondrial calcium uptake, suppresses the enzymatic activity of poly(ADP-ribose) polymerase-1 (PARP-1), and mitigates iron toxicity by scavenging free radicals.

Based on the current literature, there is no universal model for treating oncological processes; optimal concentrations, administration methods, and dosage regimens for the same antibiotic can vary depending on the associated etiology [20]. Minocycline (MC) demonstrates anti-inflammatory and immunomodulatory activity, primarily by inhibiting the activation and proliferation of T-lymphocytes, monocytes, and neutrophils, which is particularly relevant to blood cellular components [21]. The antibiotic can also neutralize free iron and increase potassium leakage from red blood cells (RBCs) [11,22].

Moreover, minocycline has been shown to enhance fibrin clot adhesion. Dentine blocks treated with MC exhibited a stronger fibrin mesh and a greater number of trapped erythrocytes, which are essential for early wound healing and connective tissue formation [23]. Given that MC can target multiple secondary damage mechanisms, treatment with this antibiotic holds significant promise for reducing harm to healthy tissues [24].

Despite the clear benefits of minocycline in treating inflammatory, oncological, and neurodegenerative conditions of various etiologies, it is essential to consider that this antibiotic has specific actions, likely due to the unique spatial organization of its molecules in different microenvironments. Given that MC can cause serious side effects due to its metabolic characteristics [25,26], studying the biophysical foundations of MC’s interaction with cellular components of the human body is of great interest [27,28].

Considering several physiological states—such as the presence of heart problems, psychiatric deviations, and oncological processes of various etiologies—erythrocytes may change their morphology as a protective reaction (potentially leading to cell death). Additionally, some chemotherapeutic drugs have a notable toxic effect on erythrocytes [29,30]. Therefore, it is crucial to work with the blood of healthy individuals. In this paper, we focus on assessing the extent of the antibiotic’s influence on the morphofunctional properties of human erythrocytes, as the direct effects of minocycline on red blood cells are still poorly understood.

## 2. Materials and Methods

### 2.1. Materials

Minocycline hydrochloride (M9511, Sigma-Aldrich (Munich, Germany)), Bovine Serum Albumin (BSAV-RO, Roche, ≥98.5%, Auckland, New Zealand), Buffer solution (1.99017 Supelco, Certipur, Sigma-Aldrich (Munich, Germany)), Acetone-2,4-DNPH solution CRM47341 Supelco, Sigma-Aldrich (Bellefonte, PA, USA), and Ethanol (1.00983, Sigma Aldrich, Supelco (Darmstadt, Germany)) were used in this research without further purification.

### 2.2. Blood Collection

Whole venous human blood was collected from 30 healthy voluntary donors (age: 18–45 years, 10 men and 20 women) according to the Good clinical practice and ethical standards for bloodletting 4 and in compliance with the Declaration of Helsinki 7th revision of 2013 and the ethical standards of the Blood Service of Voronezh Clinical Emergency Hospital No. 1. Immediately after bloodletting, blood was heparinized with the heparin level of 3 IU/mL [31].

### 2.3. Separation of Erythrocytes

The heparinized blood was centrifuged at 500× *g* for 10 min at 4 °C. Its plasma was removed, and the pellet was resuspended and washed three times with 10 mM sodium phosphate/125 mM NaCl buffer solution with pH 7.4 [32]. Washed RBCs were suspended in SPB to prepare the final stock RBC suspension with concentration of ~1 × 10^12^ cells/L.

### 2.4. Preparing RBC Suspension

In this research, we used erythrocytes from donors who had not taken any medications prior to donation. Minocycline was added to the donors’ blood after collection. The study was conducted in vitro, emphasizing the unique chemical properties of minocycline. Erythrocytes were isolated from donor blood following a standard procedure [33] after preliminary incubation with Minocycline hydrochloride (8.1 × 10^−5^ mol/L) for 1 and 24 h. This concentration was calculated based on the daily dose of the antibiotic and its molecular weight. Minocycline is available as minocycline hydrochloride in tablet and capsule forms, with dosages of 50 mg and 100 mg. Approximately 70–90% of minocycline binds to proteins in the bloodstream. This binding reduces the amount of the active drug, as only the unbound fraction of the antibiotic is microbiologically active. Minocycline is significantly more lipophilic (5 times more) than doxycycline in vivo, which allows for a higher degree of intestinal absorption. In vitro, this increased lipophilicity enables better permeability of the antibiotic through cell membranes. The plasma half-life of minocycline is about 16–18 h. Its bioavailability is excellent, ranging approximately 90–100%. The maximum concentration (Cmax) of minocycline in blood serum averages about 3.5 mcg/mL, typically achieved within 1 to 4 h after the intake of a 100 mg capsule. So, the antibiotic powder was dissolved in a predetermined volume of physiological sodium phosphate buffer and subsequently added to the erythrocyte suspension.

### 2.5. SEM

The surface architecture of donor erythrocytes was examined using scanning electron microscopy (SEM, JEOL JSM-6380LU, Tokyo, Japan). Erythrocytes were preincubated with antibiotics for 60 min. Both control and experimental samples were then fixed with a 2.5% glutaraldehyde solution at 4 °C for 1 h and dehydrated in ethanol solutions (30–90%) and acetone. The suspension was applied to an aluminum substrate and dried in a thermostat at 37 °C. To ensure electrical conductivity, a thin gold film was applied. The prepared samples were analyzed using a JSM-6380 LU scanning electron microscope (Japan) at an accelerating voltage of 20–25 kV. The surface cytoarchitecture of erythrocytes was characterized as either reversibly deformed (RD) (discocytes, discocytes with single and multiple outgrowths, “mulberry-shaped” erythrocytes) or irreversibly deformed (ID) (dome-shaped cells, spherocytes, spherocytes with surface spikes, “deflated ball” erythrocytes, and degenerative forms). Quantitative analysis was performed by calculating several indicators, including the erythrocyte transformation index—a measure of the ratio of pathological to normal erythrocytes: Transformation Index (TI) = (RD + ID)/D [33,34,35].

### 2.6. BSA Washing

Some cells were diluted in a 1:9 ratio with a 1% solution of bovine serum albumin in sodium phosphate buffer. After 10 min of washing with stirring, samples were centrifuged at 2500 rpm for 5 min. The supernatant was removed, and the pellet was resuspended in sodium phosphate buffer [36,37].

### 2.7. Spectrophotometry

The isolated hemoglobin was adjusted to an optical density of D = 0.8. The electronic absorption spectra of native and modified hemoprotein solutions were recorded in the wavelength range of 200–700 nm, spectral resolution 0.1 nm, number of replicates—4 for each donor blood sample (Shimadzu UV-2401 PC, Kyoto, Japan).

Shimadzu UV-2401PC Recording Spectrophotometer:

Spectral resolution 0.1 nm, number of replicates—4 for each donor blood sample

- Wavelength repeatability: ±0.1 nm.
- Wavelength accuracy: ±0.3 nm.
- Wavelength scanning speed: slow.
- Photometric system: Double-beam, direct ratio system with dynode feedback.
- Photometric range: Absorbance −4~5 Abs. (0.001 Abs. increments).
- Photometric accuracy ±0.002 Abs. (0~0.5 Abs.) with NIST 930D Filter (Gaithersburg, MD, USA). ±0.004 Abs. (0.5~1 Abs.) with NIST 930D Filter. ±0.3%T (0~100%T) with NIST 930D Filter.
- Photometric repeatability: ±0.001 Abs. (0~1.0 Abs.) ±0.1%T.
- Baseline flatness: Within ±0.001 Abs. (excluding noise; 2 nm slit width and SLOW wavelength scanning speed).

In this experiment, we used the special formulas for the calculation of the hemoglobin ligand forms:

[HbO_2_] = (1.7·A_577_ − 1.3·A_569_ − 0.15·A_500_)·10^−4^,

[Hb] = (−1.6·A_577_ + 2.5·A_569_ − 0.33·A_500_)·10^−4^,

[MtHb] = (0.2·A_577_ − 0.4·A_569_ + 0.33·A_500_)·10^−4^,

where [HbO_2_], [Hb], and [MtHb] are concentrations (mol/L) of hemoglobin of oxy-, deoxy-, and met-forms, and A577, A569, and A500 are absorption values at wavelengths of 577, 569, and 500 nm, respectively.

### 2.8. Molecular Docking

Computational molecular docking was employed to predict the relative positioning, orientation, and conformations of human hemoglobin molecules in the presence of the modifier, minocycline. This interaction potentially leads to the formation of a supramolecular complex. Based on structural features of erythrocyte hemoglobin, the optimal position, orientation, and conformation of the ligand (minocycline) within the protein binding site were determined [38].

The hemoglobin structure (PDB ID: 1THB; accessed on 20 August 2024, https://www.ncbi.nlm.nih.gov/Structure/pdb/1THB) was prepared for docking using Autodock Vina 1.1.2 (https://sourceforge.net/projects/autodock-vina-1-1-2-64-bit/, accessed on 20 August 2024). Solvent, buffer, and ligand atoms and coordinates were removed from the input PDB file, and the center and box parameters were manually set to ensure the hemoglobin molecule was fully enclosed within the computational space domain [39].

The minocycline structure model was created in HyperChem 8.0 (https://hyperchem.software.informer.com; accessed on 20 August 2024) and optimized sequentially using the AMBER force field, followed by PM3 (Parametric Method 3) quantum-chemical optimization. The ligand in the docking simulation had maximal conformational freedom, with rotation around all single bonds permitted. Charge distribution on the minocycline molecule and its protonation/deprotonation were handled automatically using MGLTools 1.5.6 (https://ccsb.scripps.edu/mgltools/1-5-6, accessed on 20 August 2024).

### 2.9. Statistical Analysis

Statistical data analysis was conducted using Stadia 8.0 software. The significance of differences between compared parameters was determined by Student’s *t*-test (*p* ≤ 0.05).

## 3. Results and Discussion

### 3.1. Scanning Electron Microscopy of Erythrocytes in the Presence of MC

**Figure 2:**
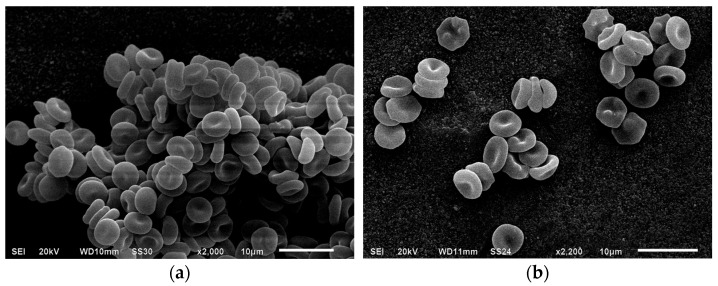
Electron micrographs of native erythrocytes (magnification ×2000, (**a**)) and those after daily incubation (magnification ×2200, (**b**)). Formation of echinocytes is observed. Explanation is in the text.

The control sample showed a typical ratio of reversibly and irreversibly transformed cell forms: discocytes accounted for 91.1 ± 1.1%, reversibly deformed forms for 97.6 ± 0.8%, and irreversibly deformed forms for 2.4 ± 0.8%. After 1 and 24 h of incubation, a decrease in the proportion of biconcave forms was observed, down to 90.2 ± 0.3% and 87.5 ± 1.6%, respectively, with no statistically significant changes in the values for reversibly deformed cell forms, the transformation index (IT), or the reversible transformation index (RTI). The incubation of intact cells in a 1% BSA solution did not alter the ratio of morphological erythrocyte variants (Figure 2).

**Figure 3:**
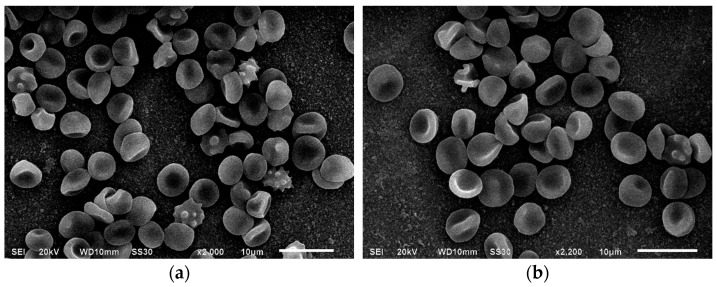
Electron micrographs of erythrocytes after a one-hour incubation with minocycline (magnification ×1500, (**a**)) and after washing with a 1% BSA solution (magnification ×1800, (**b**)). Explanation is in the text.

The modification of erythrocytes with MC for 1 h led to an increase in RTI (from 1.1 ± 0.02% to 1.2 ± 0.02% compared to control) due to the appearance of new cell forms with surface outgrowths. There was an increase in irreversibly deformed cells from 2.4 ± 0.8% to 3.5 ± 0.3% relative to control, and IT rose from 1.1 ± 0.02% to 1.2 ± 0.02% (Figure 3).

Washing the MC-modified samples with a BSA solution changed the distribution of reversibly deformed forms: discocyte levels increased from 85.2 ± 1.5% to 88.71 ± 1.0%, and the number of erythrocytes with surface outgrowths decreased from 5.2 ± 0.2% to 1.5 ± 0.7%, while the number of cells with ridges on the surface remained stable (Figure 3). In the presence of BSA, the content of spiked spherocytes decreased from 0.5 ± 0.02% to 0.1 ± 0.01%, although the number of irreversibly deformed forms remained unchanged. Daily incubation of erythrocytes with MC led to a significant increase in IT relative to the control (from 1.1 ± 0.02% to 1.2 ± 0.02%), as well as in native erythrocytes after 24 h storage (from 1.13 ± 0.03% to 1.2 ± 0.02%) and cells after 1 h incubation with MC (Table 1).

The data indicate that, compared with intact RBCs, antibiotic-modified samples exhibited a decrease in discocytes, accompanied by an increase in cells with outgrowths and ridges. However, a statistically significant increase in RTI was observed only in comparison to the control (from 1.1 ± 0.03% to 1.2 ± 0.01%). Notably, there was an increase in irreversibly deformed erythrocytes relative to the control (from 2.4 ± 0.8% to 5.1 ± 0.1%) and intact samples after daily storage (from 3.6 ± 1.1% to 5.1 ± 0.1%), primarily due to cells resembling a “deflated ball,” along with the appearance of some spherocytes and stomatocytes, which were absent in the control. Based on the data presented in the table, some of the changes may seem very small. We suppose that the case of reversible parameter decline would not be too meaningful from a biological point of view. In a real situation, a decrease in the quality of damaged red blood cells can be offset by a renewing pool of cells. However, the accelerated “aging” of cells caused by the antibiotic will potentially contribute to their elimination from the circulatory system. The presence of transformed cell forms in the bloodstream may lead to a decrease in the oxygen-transport function. “Deflated ball cells” are unable to survive rigid fluctuations in the microcirculatory bed. Thus, this might be one of the significant reasons for potential anemia during MC therapy.

**Figure 4:**
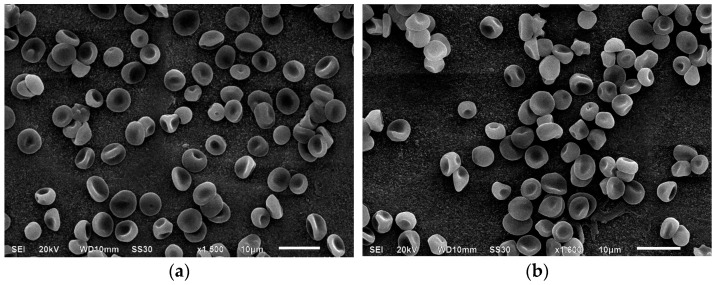
Electron micrographs of erythrocytes after a 24 h incubation with minocycline (magnification ×2000, (**a**)) and after washing with a 1% BSA solution (magnification ×2200, (**b**)).

After 24 h of incubation with MC, the transformation index increased, and RTI increased from 1.1 ± 0.02% to 1.2 ± 0.01%, mainly due to the appearance of outgrowths on biconcave cells rather than ridges. Although the IRTI index did not significantly change, there was a notable increase in irreversibly deformed cells (from 3.5 ± 0.3% to 5.1 ± 0.1%), particularly spherocytes (from 0.06 ± 0.2% to 0.6 ± 0.2%) and spiked spherocytes (from 0.5 ± 0.02% to 0.7 ± 0.02%) (Figure 4).

Washing with a 1% BSA solution reduced IT from 1.2 ± 0.02% to 1.1 ± 0.01% and RTI from 1.2 ± 0.01% to 1.1 ± 0.04%, as cells with surface outgrowths reverted to their native discocyte state. Notably, BSA had no effect on ridge-shaped cells after either 1 h or 1 day of incubation. Additionally, the number of erythrocytes with surface spikes decreased from 0.7 ± 0.02% to 0.2 ± 0.2%, while the spherocyte content remained stable.

Overall, we observed a time-dependent effect of MC on erythrocyte membranes. As shown in Figure 3 and Figure 4, incubation with MC gradually transformed discocytes with regular pallor zones into cells with ridges, suggesting that the antibiotic may accelerate erythrocyte “aging,” leading to functional cell impairment. Unlike doxycycline-modified RBCs, the antibiotic tested did not produce conditionally polymorph stomatocytes (CPS) [33]. With longer exposure times, the number and depth of membrane invaginations increased. These cytoarchitectural changes are reversible up to the stage of membrane matter loss [40,41,42]. Microvesiculation and enhanced structural rigidity of erythrocyte membranes in the presence of the antibiotic suggest its interaction with protein-lipid components, potentially affecting cell zeta potential [43]. Although BSA washing reduces the number of cells with outgrowths, it does not revert ridge-shaped and other irreversible cell forms to their original state.

### 3.2. Spectral Characteristics of Intraerythrocyte Hemoglobin Solutions

**Figure 5:**
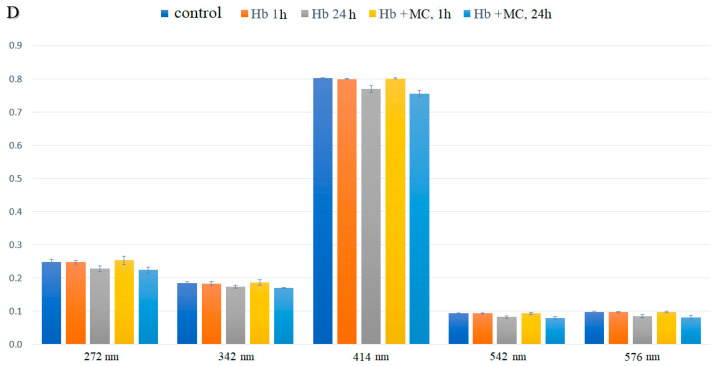
Spectral characteristics of human hemoglobin aqueous solutions in the presence of minocycline (*p* ≤ 0.05).

Analysis of the electronic absorption spectra of hemoglobin after 1 h of incubation with MC showed no statistically significant differences in the optical density values of the solutions compared to the control. However, after 24 h of incubation, optical density values decreased across three visible spectrum bands, suggesting an interaction between the hemoprotein and both the prosthetic and heme components of the molecules (Figure 5).

**Figure 6:**
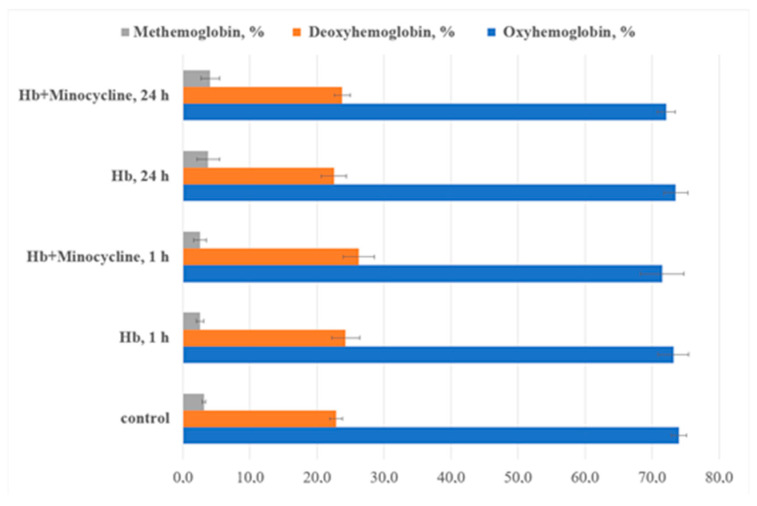
The ratio of hemoglobin ligand forms in the presence of minocycline (*p* ≤ 0.05). The major alterations are found in 24 h of MC incubation with Hb.

Statistically significant spectral differences were observed after daily incubation with the antibiotic, indicating that the most pronounced conformational changes in hemoprotein molecules require more than an hour of incubation. This may occur due to the gradual removal of steric barriers for the functional groups of the antibiotic. A decrease in optical density in the heme and globin regions of the protein suggests compaction of its molecules, which limits the penetration of oxidants into the “interior” of the protein globule. Changes in the Soret band position and other peaks during extended incubation with MC indicate a shift in the distribution of hemoglobin ligand forms toward deoxyhemoglobin (Figure 6).

A decrease in the intensity of the aromatic amino acid band was observed in the electronic absorption spectra of hemoglobin modified by MC: after 1 h of incubation, the intensity decreased by 2.3% compared to control samples, and after 24 h, by 10.4% relative to the control and 7.2% relative to native hemoglobin. A reduction in the absorption bands associated with iron porphyrin was also noted after daily modification with the drug compared to control samples. Quantitative analysis of the ligand form ratios of hemoprotein in solution with the modifier revealed no statistically significant changes compared to the control.

Thus, the antibiotic appears to stabilize the protein globule, restricting the penetration of oxidizing components into the heme region. This stabilization is supported by data showing only slight changes in optical density in the heme regions at 542 and 576 nm (Figure 5 and Figure 6).

### 3.3. Molecular Docking

**Figure 7:**
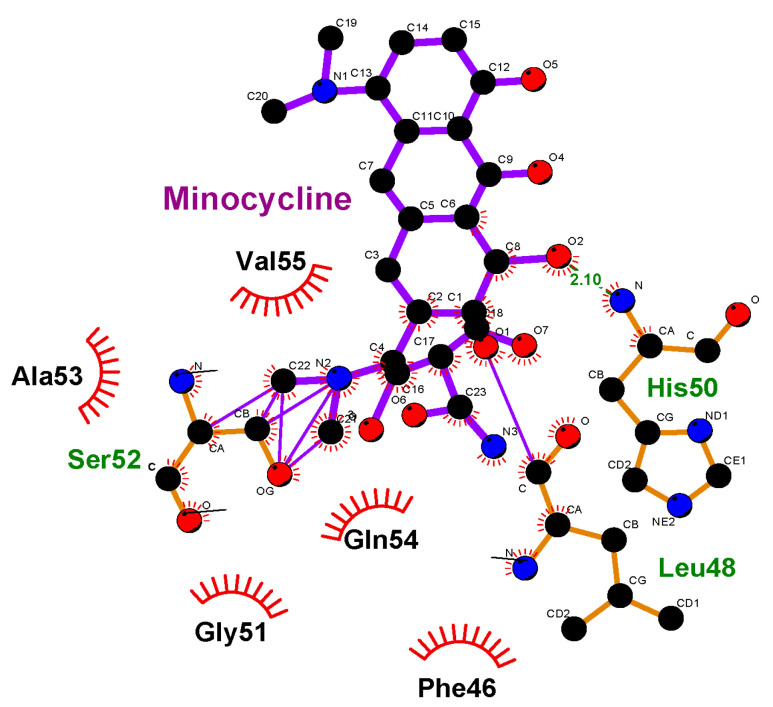
Interaction of amino acid residues in the hemoglobin with minocycline molecules. Ala53, Ser52, Phe46, Gly51, Leu48, Gln54, His50, Val55—amino acid residues of the Hb.

**Figure 8:**
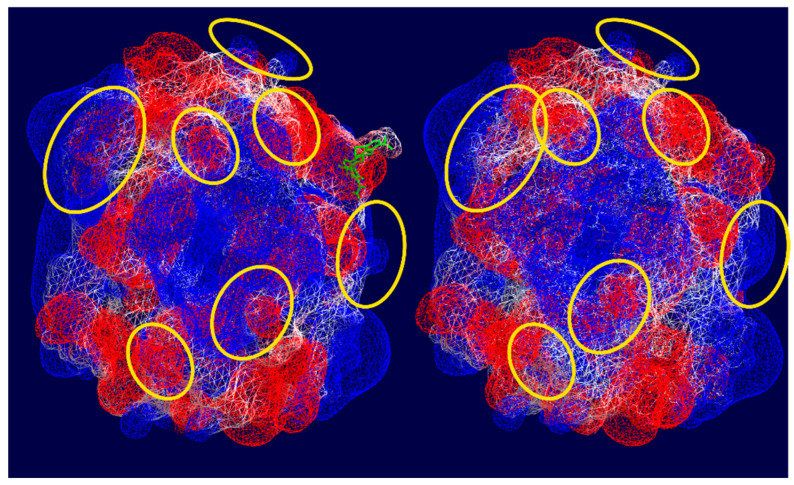
Visualization of electrostatic potential of hemoglobin surface using Swiss PDB viewer. Color representation: Blue—Positive charge, Red—Negative charge, White—Neutral charge, Green—Minocycline. Hemoglobin surface in the initial state (on high) and in the presence of minocycline (under). The molecules with the most pronounced changes in electrostatic potential are highlighted in yellow ovals.

To support the assumptions regarding the interactions between hemoglobin functional groups and MC molecules, we conducted molecular modeling of this process (Figure 7). For the calculations, a single chain of hemoprotein was used, and the localization of MC ligand binding sites on the hemoglobin surface was determined. The data indicate that structural fragments of the antibiotic orient relative to hemoglobin molecules through the dimethylamine group, forming weak bonds with Ser52. An intermolecular bond is observed between the C=O group of the antibiotic and the nitrogen of His50 (bond length = 2.10 Å), as well as with Leu48. Figure 7 shows several types of interactions, including Van der Waals and London forces. Moreover, as can be seen from Figure 8, after interaction with MC, the electrostatic potential of the hemoglobin molecule changes.

The molecular docking demonstrated proof of the potential suggestions made when the results were obtained. There is evidence that changes in the optical density of hemoglobin solutions take place in the presence of minocycline. As follows from the literature in the field, changes in the spatial organization of the molecules may lead to alterations in their rigidity and ability to bind oxygen. As shown by the molecular docking, there are several types of interactions between hemoglobin and the modifier. By hemoglobin stabilization, we understand the inability of the latter to easily change its conformation from R- to T-states.

## 4. Conclusions

Thus, minocycline has a protective effect on donor RBCs, likely by inhibiting calcium influx through their membranes. It is known that optimal gas transport by erythrocytes is achieved when they maintain a biconcave disk shape, supported by the activity of the sodium-potassium pump. Minocycline, however, promotes the appearance of more erythrocytes with ridges or in a deflated ball shape, which under in vivo conditions may slightly reduce their oxygen transport function. As the interaction time between MC and RBCs increases, the depth and number of surface invaginations also increase, although these cytoarchitectural changes in erythrocytes are largely reversible in the presence of the antibiotic.

Additionally, minocycline interacts primarily with the globin component of the hemoglobin molecule, altering its spatial conformation. The observed redistribution of electron density in hemoglobin in the presence of minocycline suggests that further research in this area is warranted. Based on the literature, it is known that the formation of atypical erythrocyte morphology is achieved via several mechanisms depending on the microenvironment of the cell. In this case, we used the donors’ blood without any pathology. The native erythrocytes were of regular and absolutely normal shape and size. The incubation with the antibiotic studied led to changes in their morphology. Due to the fact that no other trigger factors were used in this experiment, we assumed that the alterations found in the erythrocytes were the result of the modifier action. This is why we took into account all the changes observed after the preliminary storage of erythrocytes with minocycline. We were able to distinguish all the alterations between the native blood samples and those following the introduction of the antibiotic. We also noted that washing erythrocytes with a BSA solution ensures reversible alterations of the cells, which proves the unstable effects of the antibiotic on erythrocytes (as shown by scanning electron microscopy in Figure 3 and Figure 4). Based on data obtained through spectrophotometry and molecular docking methods, the nature of the interactions between hemoglobin molecules and the antibiotic involves weak types of binding, such as Van der Waals forces, hydrophobic interactions, or London dispersion forces. The data suggest that younger erythrocytes are more resistant to the effects of the modifier, and these effects diminish over time.

Based on the chemical structure of minocycline, it is able to have potential interactions with intraerythrocyte components, like the cytoskeleton (actin/spectrin links) and hemoglobin. The fact of hemoglobin interaction with the modifier was proven by spectrophotometry. In our previous studies, it was demonstrated that antibiotics can be bound to carboxylic and amino groups of the hemoglobin, with its histidine, arginine, lysine, and tyrosine. Such interactions affect the spatial conformation of the molecule, leading to changes in the ability to bind oxygen. In this article, we concentrated on the effects of minocycline on the structural and—finally—functional changes in the hemoglobin. Taking into account that high lipophilicity increases tissue penetration of minocycline, it can easily penetrate through the erythrocyte membrane. This is why we observed an optical density shift when performing spectrophotometry of the hemoglobin solution taken from erythrocytes after their preliminary incubation with the modifier. We do not think that minocycline may change the viscoelasticity of erythrocytes’ membrane too much, because in the case of the concentration we used, the ratio between antibiotic and hemoglobin molecules was 1:3, respectively. In other words, the data obtained indicates the «aging» of erythrocytes in the presence of minocycline. In this study, we did not perform any tests to check the change in the membrane viscoelasticity directly. But we understand that the extension of the experimental setup is needed. This could help to confirm the assumptions made in the article with confidence. We will examine some of these issues in more detail in several subsequent articles.

So, in our opinion, the change in cell shape occurs due to the interaction of the antibiotic with the erythrocytes’ cytoskeleton. Based on the data obtained in this study, further exploration of this phenomenon (e.g., in vivo tests, studies in patients undergoing minocycline therapy, and comparison with other antibiotics) is needed to minimize the side effects of the antibiotic studied.

## Figures and Tables

**Table 1:** Cytoarchitectonics of erythrocytes modified with minocycline.

Indices, %	Control	Incubation with MC, 1 h	Incubation with MC + BSA, 1 h	Incubation with MC, 24 h	Incubation with MC + BSA, 24 h
Discocytes	91.1 ± 1.1	85.2 ± 1.5	88.7 ± 1.1	81.4 ± 0.9	85.9 ± 1.2
Echinocytes	3.5 ± 0.5	5.2 ± 0.2	1.5 ± 0.7	7.4 ± 1.4	4.0 ± 0.9
Discocytes with a “ridge”	3.1 ± 0.3	6.1 ± 0.3	6.0 ± 0.6	6.2 ± 0.4	5.6 ± 0.9
Reversibly deformed	97.6 ± 0.8	96.4 ± 0.6	96.2 ± 0.6	95.0 ± 0.6	95.4 ± 0.7
“deflated ball”	0.2 ± 0.02	1.1 ± 0.9	1.2 ± 0.4	1.6 ± 0.1	1.6 ± 0.4
“dome-shaped”	1.2 ± 1.07	1.2 ± 0.5	1.8 ± 0.6	1.3 ± 0.2	1.2 ± 0.4
Spherocytes	0.0 ± 0.0	0.06 ± 0.02	0.09 ± 0.0	0.5 ± 0.2	0.5 ± 0.1
Stomatocytes	0.0 ± 0.0	0.02 ± 0.01	0.01 ± 0.0	0.1 ± 0.0	0.2 ± 0.0
Degenerative forms	0.5 ± 0.0	0.6 ± 0.2	0.6 ± 0.1	0.7 ± 0.3	0.7 ± 0.2
Spherocytes with spikes	0.4 ± 0.1	0.5 ± 0.02	0.1 ± 0.0	0.7 ± 0.02	0.2 ± 0.1
Irreversibly deformed	2.4 ± 0.8	3.5 ± 0.3	3.7 ± 0.5	5.1 ± 0.1	4.5 ± 0.2
Transformation index	1.1 ± 0.02	1.2 ± 0.02	1.1 ± 0.01	1.2 ± 0.02	1.2 ± 0.01
Index of reversible transformation	1.1 ± 0.03	1.1 ± 0.02	1.1 ± 0.01	1.2 ± 0.01	1.1 ± 0.04
Index of irreversible transformation	0.03 ± 0.02	0.04 ± 0.01	0.04 ± 0.02	0.06 ± 0.03	0.05 ± 0.01

## Data Availability

The data is unavailable due to privacy or ethical restrictions.

## Abbreviations

The following abbreviations are used in this manuscript:

TGF-β	Transforming growth factor beta
NF-κB	Nuclear factor kappa-light-chain-enhancer of activated B cells
IKKα/β	IkappaB kinase
HIF-1α	Hypoxia-inducible factor 1-alpha
VEGF	Vascular endothelial growth factor
eIF2α	Eukaryotic initiation factor 2
NMDA	*N*-methyl-D-aspartate receptors
TNF-α	Tumor necrosis factor-alpha
mTOR	Mechanistic target of rapamycin
IL	Interleukin
MCP-1	Monocyte chemoattractant protein 1
MIP-1α	Macrophage inflammatory protein
